# Mitochondrial Immunometabolism in Sepsis: From Oxidative Stress and mtDAMP Signaling to Biomarker-Guided Therapy

**DOI:** 10.3390/ijms27135918

**Published:** 2026-06-30

**Authors:** Minsoo Kim, Phyu Phyu Khin, Hyeran Jung, Chang Woo Chae, Byeong Hwa Jeon, Cuk-Seong Kim

**Affiliations:** Department of Physiology & Medical Science, College of Medicine, Chungnam National University, Daejeon 35015, Republic of Korea; g4qsol@naver.com (M.K.); phyuphyukim92@gmail.com (P.P.K.); phdjuliet@gmail.com (H.J.); chae.cw@cnu.ac.kr (C.W.C.); bhjeon@cnu.ac.kr (B.H.J.)

**Keywords:** sepsis, mitochondria, oxidative stress, mitochondrial DNA, mitophagy, immunometabolism, organ dysfunction, biomarkers, mitochondria-targeted therapy

## Abstract

Sepsis is a life-threatening syndrome characterized by a dysregulated host response to infection and progressive organ dysfunction. Although early antimicrobial therapy, source control, hemodynamic resuscitation, and organ support remain the foundations of care, these approaches do not directly reverse the cellular mechanisms that connect systemic inflammation to multi-organ failure. Mitochondrial dysfunction has emerged as a central mechanism linking impaired oxygen utilization, oxidative and nitrosative stress, immune-cell metabolic reprogramming, inflammatory amplification, and organ injury. During sepsis, inflammatory mediators, nitric oxide, microcirculatory abnormalities, calcium dysregulation, and metabolic stress converge on mitochondria, impairing oxidative phosphorylation and promoting mitochondrial reactive oxygen species/reactive nitrogen species (ROS/RNS) generation. When mitochondrial quality-control programs, including fission, fusion, mitophagy, and mitochondrial biogenesis, fail to restore network integrity, damaged mitochondria accumulate and become persistent sources of oxidative stress and danger signals. Mitochondrial damage-associated molecular patterns, particularly mitochondrial DNA, oxidized mitochondrial DNA, cardiolipin, ATP, and N-formyl peptides, activate innate immune pathways such as TLR9-MyD88-NF-kappaB, the NLRP3 inflammasome, and cGAS-STING signaling. In parallel, mitochondrial metabolism shapes macrophage activation, neutrophil function, T-cell competence, pyruvate-lactate handling through the pyruvate dehydrogenase complex, and the transition between hyperinflammation and immunosuppression. Clinical translation remains challenging because sepsis is biologically heterogeneous and mitochondrial dysfunction is dynamic, tissue-specific, and influenced by disease stage. This review synthesizes current knowledge on mitochondrial dysfunction in sepsis, emphasizing oxidative and nitrosative stress, mitochondrial quality control, mitochondrial damage-associated molecular pattern (DAMP) signaling, immunometabolism, organ-specific injury, candidate biomarkers, clinical translational strategies for mitochondria-targeted therapy, and future approaches based on multi-omics and artificial intelligence-assisted patient stratification. We argue that future therapeutic development should move beyond nonspecific antioxidant supplementation toward time-sensitive, phenotype-informed, and biomarker-guided mitochondrial medicine.

## 1. Introduction

Sepsis remains a major global health challenge and a leading cause of death, disability, and resource use in acute care and intensive care medicine. The Sepsis-3 consensus defined sepsis as life-threatening organ dysfunction caused by a dysregulated host response to infection, and operationalized organ dysfunction using an acute increase in the Sequential Organ Failure Assessment score [1,2]. Recent global burden estimates have reinforced the scale of the problem. A 2025 Global Burden of Disease analysis estimated 166 million sepsis cases and 21.4 million all-cause sepsis-related deaths worldwide in 2021, while earlier widely cited estimates for 2017 reported 48.9 million cases and 11.0 million sepsis-related deaths [3,4]. These estimates differ in methodology and case definition, but together highlight that sepsis remains among the most consequential syndromes in global health.

Current sepsis management is built around early recognition, prompt antimicrobial therapy, source control, hemodynamic resuscitation, vasopressors when needed, respiratory and renal support, and prevention of secondary complications [5,6]. These interventions are essential and lifesaving, but they are not designed to directly reverse the cellular and subcellular processes that connect infection to organ failure. Many clinical trials targeting single inflammatory mediators have failed to produce durable benefit, reflecting the biological heterogeneity of sepsis and the coexistence of hyperinflammation, immune suppression, endothelial dysfunction, metabolic reprogramming, and impaired tissue oxygen utilization [7,8]. Consequently, attention has shifted from viewing sepsis as only an uncontrolled inflammatory response to understanding it as a dynamic host-response syndrome in which metabolism and immunity are tightly integrated.

Mitochondria sit at the center of this integration. Beyond their canonical role in ATP production through oxidative phosphorylation, mitochondria regulate redox homeostasis, calcium handling, apoptosis, innate immune signaling, inflammasome activation, and immune-cell fate. In sepsis, inflammatory mediators, nitric oxide, hypoxia-like signaling, microcirculatory dysfunction, and metabolic substrate shifts converge on mitochondria. The result may be impaired electron transport chain activity, ATP depletion, mitochondrial membrane potential loss, calcium overload, mitochondrial permeability transition, and excessive production of mitochondrial reactive oxygen species/reactive nitrogen species (ROS/RNS). This process contributes to the concept of cytopathic hypoxia, in which oxygen delivery may be adequate but cellular oxygen utilization is impaired [9,10,11,12,13,14].

The original working concept for this review focused on mitochondria as the primary site of ATP generation and an important source of reactive oxygen species, proposing that sepsis-induced reactive oxygen species (ROS) overproduction disrupts mitochondrial function, contributes to organ damage, and may require mitochondria-targeted rather than systemic antioxidant therapy. The present version preserves that core concept but expands it into a broader framework: mitochondrial dysfunction in sepsis is not merely a consequence of oxidative injury, but a driver of immunometabolic failure, mitochondrial damage-associated molecular pattern (DAMP) release, inflammatory amplification, and multi-organ dysfunction.

**Figure 1:**
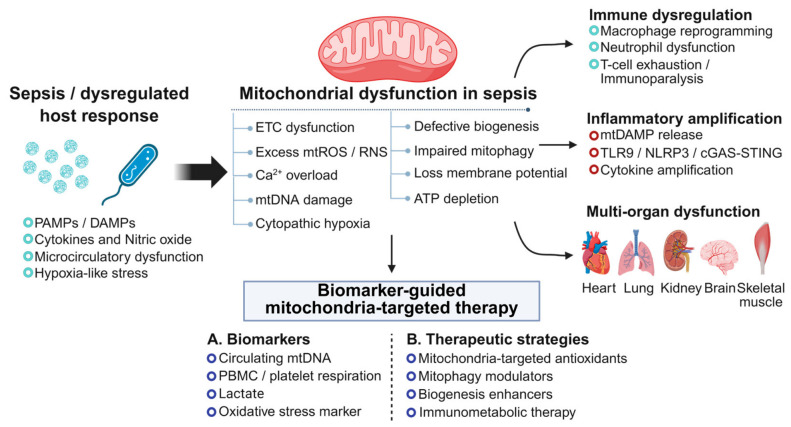
Mitochondria-centered model of sepsis pathophysiology. In sepsis, infection-driven inflammatory and metabolic stress converge on mitochondria, causing impaired oxidative phosphorylation, excessive mitochondrial ROS/RNS, ATP depletion, calcium dysregulation, mitochondrial membrane potential loss, and defective mitochondrial quality control. Damaged mitochondria then amplify inflammation through mitochondrial DAMP release, reshape immune-cell metabolism, and contribute to organ dysfunction. These features support the development of mitochondria-targeted and biomarker-guided therapeutic strategies. The figure was created with BioRender.com (accessed on 25 May 2026).

In this review, we synthesize current knowledge on mitochondrial dysfunction in sepsis, focusing on oxidative and nitrosative stress, mitochondrial quality control, mitochondrial DAMP signaling, immunometabolic dysregulation, organ-specific injury, candidate biomarkers, and mitochondria-targeted therapeutic strategies. We propose a mitochondria-centered framework in which mitochondrial injury acts not only as a consequence of septic inflammation but also as an active driver of immune failure, inflammatory amplification, and organ dysfunction (Figure 1).

## 2. Mitochondrial Dysfunction as a Central Mechanism in Sepsis

Mitochondria are essential for cellular energy homeostasis. Electrons derived from nutrient oxidation enter the electron transport chain through complexes I and II and are transferred through complexes III and IV, generating a proton gradient across the inner mitochondrial membrane. ATP synthase uses this electrochemical gradient to phosphorylate ADP to ATP. Under physiological conditions, mitochondrial respiration is coupled to ATP demand, whereas antioxidant and repair systems limit reactive oxygen species accumulation and counteract oxidative damage. Under septic conditions, these protective systems may become insufficient. Cells are exposed to inflammatory cytokines, pathogen-associated molecular patterns, damage-associated molecular patterns, nitric oxide, catecholamines, altered substrate availability, and fluctuating oxygen delivery. These insults may impair respiratory chain complexes, disrupt the proton gradient, increase proton leak, reduce ATP generation, and promote mitochondrial permeability transition [10,11,12,13,14,15].

The relationship between mitochondrial function and organ failure was highlighted by early clinical and translational studies showing that mitochondrial dysfunction correlates with severity and outcomes in septic shock [10]. Experimental endotoxemia and infection models similarly demonstrate mitochondrial structural damage, reduced respiratory activity, and impaired oxidative phosphorylation in multiple tissues [11,12]. These observations support a model in which organ dysfunction may develop even without extensive cell death. In other words, cells may remain structurally present but metabolically unable to sustain normal specialized functions. This state has been described as bioenergetic failure or cytopathic hypoxia [9,10,13].

Cytopathic hypoxia does not imply that oxygen delivery is irrelevant. Shock, microvascular shunting, endothelial dysfunction, anemia, hypoxemia, and edema can all limit oxygen delivery. However, the concept emphasizes that tissue oxygen utilization may be impaired even when macrocirculatory variables appear corrected. Nitric oxide can reversibly inhibit cytochrome c oxidase, while peroxynitrite and other reactive species can cause more persistent damage to respiratory chain components. Reduced ATP availability affects ion pumps, barrier function, contractility, phagocytosis, antigen presentation, and protein synthesis, making mitochondrial dysfunction a plausible common denominator for organ failure across tissues.

Importantly, mitochondrial changes during sepsis are not uniformly suppressive. Some circulating immune-cell populations show reduced respiration in certain studies, whereas others demonstrate increased basal respiration, proton leak, or compensatory activation depending on disease phase, cell type, substrate conditions, and assay methodology [16,17]. This heterogeneity may reflect distinct biological states: adaptive metabolic activation, mitochondrial stress, inefficient respiration, or true respiratory failure. Therefore, mitochondrial dysfunction should not be defined only as decreased oxygen consumption. Instead, it should be viewed as a loss of appropriate mitochondrial regulation, in which respiration, ATP production, redox control, dynamics, quality control, and immune signaling become mismatched to cellular needs.

This broader definition is clinically important. It explains why simple correction of oxygen delivery or nonspecific suppression of inflammation may be insufficient. It also suggests that mitochondrial therapies must be matched to the specific mitochondrial phenotype: excessive mtROS production, impaired biogenesis, defective mitophagy, membrane instability, metabolic paralysis, or immune-cell mitochondrial overactivation.

## 3. Oxidative and Nitrosative Stress in Septic Mitochondria

Oxidative stress has long been recognized as a major component of sepsis pathobiology. In the mitochondrial context, reactive oxygen species arise when electrons leak from redox centers within the electron transport chain and reduce molecular oxygen to superoxide. Contrary to older simplified descriptions that attributed superoxide production mainly to complex IV, current mitochondrial biology identifies complexes I and III, as well as several matrix and membrane-associated dehydrogenases, as important sources of mitochondrial superoxide and hydrogen peroxide under stress conditions [18,19,20]. Physiological ROS participate in redox signaling, antimicrobial defense, and adaptive stress responses. In sepsis, however, inflammatory stimulation, substrate overload, respiratory chain inhibition, ischemia–reperfusion-like events, and antioxidant depletion may drive excessive mitochondrial ROS production.

Nitrosative stress develops in parallel. Inducible nitric oxide synthase can generate large amounts of nitric oxide in immune, vascular, and parenchymal cells. Nitric oxide may reversibly compete with oxygen at complex IV, thereby inhibiting mitochondrial respiration. When nitric oxide reacts with superoxide, it forms peroxynitrite, a potent oxidant capable of nitrating proteins, damaging lipids, modifying iron-sulfur centers, impairing respiratory complexes, and damaging mitochondrial DNA [21]. Thus, ROS and reactive nitrogen species interact to create a feed-forward cycle: respiratory inhibition increases electron leak, electron leak increases superoxide, superoxide reacts with nitric oxide to form peroxynitrite, and peroxynitrite further impairs mitochondrial enzymes.

Mitochondrial DNA is particularly vulnerable to oxidative injury. It is located near the inner mitochondrial membrane, lacks protective histones, and encodes essential components of oxidative phosphorylation. Oxidative mtDNA damage may impair transcription of respiratory-chain subunits and promote further respiratory dysfunction. Damaged or oxidized mtDNA may also become immunostimulatory when released into the cytosol or extracellular space. In this sense, oxidative stress is not only a marker of cellular injury but also a mechanism that transforms mitochondria into inflammatory signaling platforms.

Cells counterbalance oxidative stress through antioxidant defense systems. Manganese superoxide dismutase converts mitochondrial superoxide to hydrogen peroxide. Hydrogen peroxide is then detoxified by glutathione peroxidases, peroxiredoxins, thioredoxin-dependent systems, and catalase in relevant compartments. Sepsis may alter this network through substrate depletion, selenium deficiency, reduced glutathione availability, impaired NADPH-dependent regeneration, or enzyme inactivation. Clinical and experimental studies have generally described reduced antioxidant capacity, including decreased superoxide dismutase, catalase, glutathione peroxidase activity, and reduced thiol/glutathione status, together with increased lipid and protein oxidation markers such as malondialdehyde and protein carbonyls in sepsis, although the direction and magnitude of these changes vary with timing, compartment, severity, and assay method [13,14]. However, redox biomarkers are difficult to interpret because they vary by compartment, timing, illness severity, and assay method.

The central translational lesson is that oxidative stress in sepsis is compartmentalized. Systemic antioxidant supplementation may not reach the mitochondria at sufficient concentration, may be given at the wrong disease phase, may interfere with beneficial antimicrobial ROS signaling, or may be applied to patients whose dominant pathophysiology is not oxidative injury. These limitations help explain why conventional antioxidant therapy has produced inconsistent clinical results. A more rational approach is to consider mitochondrial redox stress together with mitochondrial quality control and immune phenotype.

**Figure 2:**
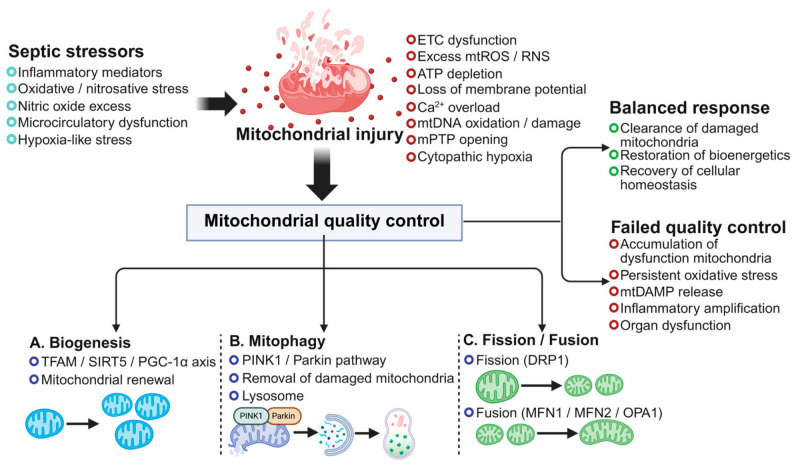
Mitochondrial injury and quality-control imbalance during sepsis. Sepsis-associated oxidative and nitrosative stress impairs electron transport chain function, decreases ATP production, and promotes mitochondrial injury. In response, cells engage mitochondrial quality-control mechanisms including fission, fusion, mitophagy, and biogenesis. When these adaptive programs are insufficient, dysfunctional mitochondria accumulate, leading to sustained oxidative stress, mitochondrial DAMP release, and organ dysfunction. The figure was created with BioRender.com (accessed on 25 May 2026).

Figure 2 summarizes the transition from septic stressors to mitochondrial injury and illustrates how mitochondrial quality-control pathways, including biogenesis, mitophagy, and fission/fusion, determine whether cells recover bioenergetic homeostasis or progress toward persistent dysfunction.

## 4. Mitochondrial Quality Control: Fission, Fusion, Mitophagy, and Biogenesis

Mitochondrial injury does not inevitably lead to irreversible cellular dysfunction. Cells possess quality-control programs that remodel, remove, and replace damaged mitochondria. These programs include mitochondrial fission and fusion, mitophagy, proteostasis, mitochondrial unfolded protein responses, and mitochondrial biogenesis. Sepsis challenges these adaptive mechanisms by increasing mitochondrial injury while simultaneously altering the signaling pathways that coordinate repair [15,22,23,24,25,26]. Whether a cell recovers or progresses toward dysfunction may depend on the balance between mitochondrial damage and mitochondrial quality control.

### 4.1. Dynamic Remodeling of the Mitochondrial Network

Fission and fusion regulate mitochondrial morphology, distribution, and functional complementation. Fission, mediated largely by dynamin-related protein 1, can isolate damaged mitochondrial segments and facilitate mitophagy. Excessive or prolonged fission, however, may fragment the mitochondrial network, reduce respiratory efficiency, and promote apoptosis or inflammasome activation. Fusion, mediated by mitofusin 1, mitofusin 2, and optic atrophy 1, allows mitochondrial content mixing and preservation of membrane potential. In sepsis, inflammatory signaling and oxidative stress may shift the balance toward fragmentation in some tissues, while compensatory fusion or remodeling may occur in others. The net effect is likely tissue- and time-dependent.

Mitochondrial dynamics also intersect with immune signaling. Fragmented mitochondria may generate more ROS, release mtDNA more readily, and interact with inflammasome components. Conversely, preserved mitochondrial networks may support oxidative metabolism, tissue repair, and immune resolution. Therefore, mitochondrial dynamics are not merely structural phenomena; they influence redox biology, cell death susceptibility, and inflammatory tone.

### 4.2. Defective Mitophagy in Sepsis

Mitophagy selectively removes damaged mitochondria through autophagy-lysosome pathways. The PINK1/Parkin pathway is among the best characterized: loss of mitochondrial membrane potential stabilizes PINK1 on the outer mitochondrial membrane, recruits Parkin, and promotes ubiquitination of mitochondrial proteins that are recognized by autophagy adaptors. Receptor-mediated pathways involving BNIP3, NIX, FUNDC1, and other proteins can also target mitochondria for degradation [22,23,24,25].

During sepsis, mitophagy may be protective by limiting mtROS production and preventing release of mitochondrial DAMPs. Experimental models suggest that restoring mitophagy can reduce inflammation and organ injury, while impaired mitophagy permits accumulation of dysfunctional mitochondria [15,22,23,24,25]. However, mitophagy is not universally beneficial in all contexts. Excessive mitophagy without adequate biogenesis may reduce mitochondrial mass and worsen bioenergetic capacity. Thus, mitophagy must be interpreted as part of a broader quality-control program rather than as an isolated therapeutic target.

### 4.3. Mitochondrial Biogenesis as a Recovery Program

Mitochondrial biogenesis replenishes mitochondrial mass and supports recovery after injury. Key regulators include AMPK, SIRT1, SIRT3, PGC-1alpha, NRF1, NRF2, and mitochondrial transcription factor A. These pathways coordinate nuclear and mitochondrial gene expression, antioxidant defenses, respiratory-chain assembly, and mtDNA replication [25,26]. In sepsis, mitochondrial biogenesis may represent an adaptive response to restore energy production during recovery. Failure to mount this response may contribute to persistent organ dysfunction, immune paralysis, and prolonged weakness.

The therapeutic implication is that successful mitochondrial medicine may require dual action: removal of damaged mitochondria and restoration of functional mitochondrial networks. A drug that reduces mtROS without improving mitophagy or biogenesis may blunt oxidative biomarkers but fail to restore tissue function. Conversely, stimulating biogenesis without controlling ongoing oxidative injury may expand a dysfunctional network. Future strategies should therefore evaluate mitochondrial quality control as an integrated system.

The major mitochondrial mechanisms implicated in sepsis, including impaired oxidative phosphorylation, mtROS/RNS excess, calcium dysregulation, defective mitophagy, insufficient biogenesis, mtDAMP release, and immunometabolic failure, are summarized in Table 1.

## 5. Mitochondrial DAMP Signaling and Inflammatory Amplification

One of the most important advances in mitochondrial biology is the recognition that damaged mitochondria can act as danger-signal platforms. Mitochondria evolved from bacterial ancestors and retain bacterial-like molecular features, including circular unmethylated CpG-rich DNA and N-formyl peptides. When mitochondrial membranes are disrupted or when mitophagy fails, mitochondrial components may enter the cytosol or extracellular space and function as mitochondrial damage-associated molecular patterns [28,29,31,32,33,34,35,36,37].

Mitochondrial DNA is the best studied mitochondrial DAMP. Cytosolic mtDNA can activate cGAS, leading to production of cyclic GMP-AMP, activation of STING, phosphorylation of TBK1 and IRF3, and induction of type I interferon responses [34,35]. Extracellular or endosomal mtDNA can activate TLR9, leading through MyD88 to NF-kappaB activation and inflammatory cytokine production [36]. Oxidized mtDNA may be particularly immunostimulatory and has been implicated in NLRP3 inflammasome activation [28,29]. These pathways provide a mechanistic bridge between mitochondrial injury and systemic inflammation.

The NLRP3 inflammasome is especially relevant in sepsis. Mitochondrial ROS, oxidized mtDNA, cardiolipin exposure, potassium efflux, lysosomal injury, and metabolic stress can contribute to NLRP3 activation. Activated NLRP3 promotes caspase-1 activation, maturation of IL-1beta and IL-18, and pyroptosis. Pyroptotic cell death can further release DAMPs and amplify inflammation. Autophagy and mitophagy normally restrain this process by removing damaged mitochondria and limiting mtDNA release; when these pathways are impaired, inflammasome activation may persist [28,29,37].

Cardiolipin, a phospholipid normally enriched in the inner mitochondrial membrane, may externalize to the outer mitochondrial membrane during stress and interact with immune signaling complexes. N-formyl peptides can activate formyl peptide receptors on neutrophils, promoting chemotaxis and activation. ATP released from damaged cells can signal through purinergic receptors and contribute to inflammasome activation. Cytochrome c release participates in apoptotic signaling and may indirectly shape immune responses through cell death and secondary DAMP release.

Mitochondrial DAMP signaling also intersects with endothelial injury, neutrophil activation, and immunothrombosis. Neutrophil extracellular traps can trap pathogens but may also damage endothelium, expose histones and DNA, activate coagulation, and worsen organ injury [38,39]. Mitochondrial components can contribute to this process directly by activating neutrophils or indirectly by increasing cytokine and endothelial activation. Therefore, mitochondrial damage can propagate inflammation beyond the initially infected tissue.

**Figure 3:**
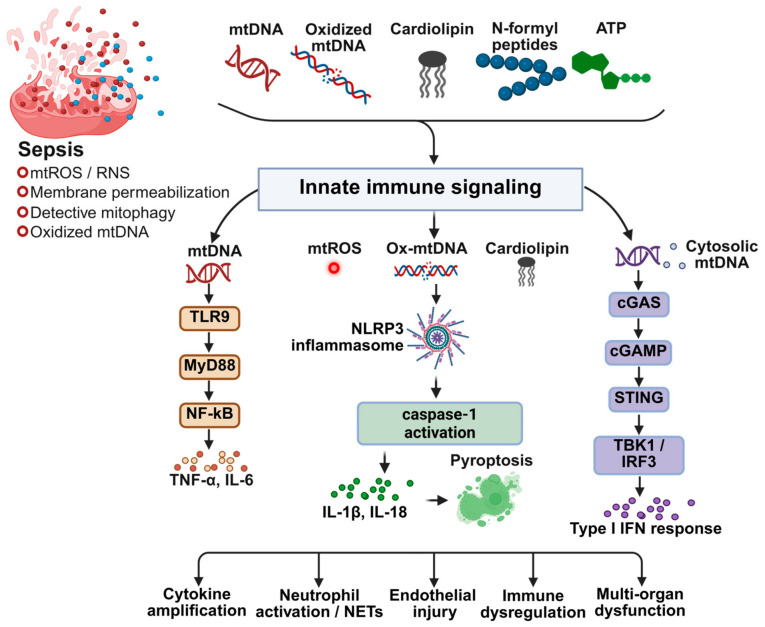
Mitochondrial DAMP release and innate immune signaling in sepsis. During sepsis, damaged mitochondria release mitochondrial damage-associated molecular patterns, including mitochondrial DNA, oxidized mitochondrial DNA, cardiolipin, N-formyl peptides, and ATP. These signals activate innate immune pathways such as TLR9-MyD88-NF-kappaB, the NLRP3 inflammasome, and cGAS-STING, resulting in cytokine amplification, inflammasome activation, pyroptosis, neutrophil activation, endothelial injury, immune dysregulation, and multi-organ dysfunction. The figure was created with BioRender.com (accessed on 25 May 2026).

This framework changes how mitochondrial dysfunction should be interpreted in sepsis. Damaged mitochondria are not silent victims of inflammation. They are active amplifiers that can convert metabolic stress into innate immune activation. Figure 3 illustrates the release and sensing of mitochondrial DAMPs in sepsis, showing how mtDNA, oxidized mtDNA, cardiolipin, N-formyl peptides, and ATP activate TLR9, NLRP3 inflammasome, and cGAS-STING signaling pathways to amplify inflammation and organ injury.

### Clinical Relevance and Therapeutic Implications of mtDAMP Signaling

The clinical relevance of mitochondrial DAMP signaling is increasingly supported by studies measuring circulating cell-free mtDNA in patients with sepsis and septic shock. Plasma or serum mtDNA concentrations are generally higher in sepsis than in non-septic controls and tend to increase further in septic shock. Several clinical studies have reported associations between circulating mtDNA and SOFA score, APACHE II score, lactate, inflammatory markers, organ dysfunction, and mortality, suggesting that mtDNA can function as both a biomarker of mitochondrial injury and a mediator of innate immune activation [32,33,45,46,47]. These observations do not yet establish mtDNA as a stand-alone diagnostic or prognostic test, because assays differ in sample processing, target genes, normalization, and distinction between cell-free, vesicle-associated, and cellular mtDNA. Nevertheless, they strengthen the concept that persistent mtDAMP release is clinically relevant rather than only experimentally interesting.

The therapeutic implication is that mtDAMP pathways may be targeted at different levels. Upstream approaches include preservation of mitochondrial membrane integrity, reduction in excessive mtROS, and restoration of mitophagy to prevent mtDNA leakage. Downstream approaches include inhibition of TLR9, NLRP3 inflammasome signaling, cGAS-STING activation, purinergic signaling, or formyl-peptide receptor pathways [28,29,31,32,33,34,35,36,37]. At present, these strategies remain largely preclinical in sepsis, and no mtDNA-specific neutralizing antibody or receptor antagonist has become standard therapy for septic patients. Importantly, complete blockade of mtDAMP sensing could impair host defense and pathogen clearance. Therefore, future development should emphasize stage-specific inhibition of excessive or persistent mtDAMP signaling rather than broad immunosuppression.

## 6. Mitochondrial Immunometabolism and Immune Dysregulation in Sepsis

Sepsis is characterized by dynamic immune dysregulation rather than a simple linear progression from inflammation to immunosuppression. Hyperinflammatory and immunosuppressive features may coexist in the same patient, and different immune-cell populations may occupy distinct metabolic states. Mitochondria are central to this immunometabolic complexity because immune-cell activation requires coordinated changes in ATP production, biosynthesis, redox signaling, substrate utilization, and epigenetic regulation [40,41,42,43,44].

Macrophages illustrate this principle. In simplified models, inflammatory macrophage activation is associated with increased glycolysis, disrupted tricarboxylic acid cycle flux, succinate accumulation, nitric oxide production, and ROS-dependent signaling. Reparative or resolution-associated macrophage states are often linked to oxidative phosphorylation, fatty acid oxidation, intact mitochondrial respiration, and mitochondrial biogenesis. However, septic tissues rarely fit a strict M1/M2 binary model. Macrophages can display hybrid phenotypes, organ-specific transcriptional programs, tolerance-like states, and time-dependent transitions. Mitochondrial function influences this plasticity by regulating metabolites, ROS, NAD+/NADH balance, and inflammatory signaling [40,41,42].

In early sepsis, mitochondrial ROS and metabolic rewiring may support antimicrobial and inflammatory responses. Excessive or persistent mitochondrial stress, however, can impair phagocytosis, reduce antigen presentation, increase inflammasome activation, or promote immunoparalysis. A macrophage with impaired oxidative phosphorylation may produce inflammatory mediators but fail to clear pathogens effectively or support tissue repair. Conversely, excessive metabolic suppression may contribute to secondary infection risk and persistent critical illness.

Neutrophils are traditionally considered glycolysis-dependent because they operate in hypoxic inflammatory environments and contain relatively few mitochondria. Nevertheless, mitochondria influence neutrophil lifespan, apoptosis, ROS signaling, calcium handling, and neutrophil extracellular trap (NET) formation. Dysregulated NET formation in sepsis may contribute to endothelial injury, microvascular thrombosis, and organ dysfunction [38,39]. Mitochondrial DAMPs and mitochondrial ROS can amplify neutrophil activation, while delayed neutrophil apoptosis may prolong tissue injury.

T cells are also metabolically vulnerable in sepsis. Effective T-cell activation, proliferation, memory formation, and effector function require coordinated glycolysis and mitochondrial metabolism. Sepsis-associated lymphopenia, T-cell exhaustion, checkpoint pathway activation, apoptosis, and impaired cytokine responses are well described [43,44]. Mitochondrial dysfunction may contribute by reducing spare respiratory capacity, increasing oxidative stress, altering mitochondrial membrane potential, and limiting metabolic flexibility. These changes may help explain why some patients transition from early inflammatory shock to prolonged immunosuppression and vulnerability to secondary infection.

The immunometabolic view has therapeutic implications. A mitochondria-targeted drug may have different effects depending on whether it is given during early hyperinflammation, pathogen clearance, immune paralysis, or recovery. Suppressing mitochondrial ROS too early may impair antimicrobial signaling, while restoring mitochondrial function later may enhance immune competence. This temporal complexity strengthens the argument for biomarker-guided and phenotype-specific mitochondrial interventions.

### 6.1. Pyruvate Dehydrogenase Complex Dysfunction and Sepsis-Associated Hyperlactatemia

A key immunometabolic mechanism that deserves explicit consideration is dysfunction of the mitochondrial pyruvate dehydrogenase complex (PDC). PDC links cytosolic glycolysis to mitochondrial oxidation by converting pyruvate into acetyl-CoA for entry into the tricarboxylic acid cycle. During sepsis, inflammatory signaling, hypoxia-like responses, nitric oxide, substrate stress, and activation of pyruvate dehydrogenase kinases can phosphorylate and inhibit PDC. As a result, pyruvate is diverted toward lactate production by lactate dehydrogenase, while mitochondrial acetyl-CoA supply, NADH generation, TCA cycle flux, and oxidative phosphorylation are reduced [48,49]. This mechanism helps explain why hyperlactatemia in sepsis should not be interpreted only as a marker of anaerobic hypoperfusion. Lactate may also reflect impaired mitochondrial pyruvate entry, accelerated glycolysis driven by adrenergic and inflammatory stimulation, altered hepatic clearance, and immune-cell metabolic reprogramming.

PDC dysfunction also links metabolism to inflammation. Reduced pyruvate oxidation can promote accumulation of immunologically active metabolites such as succinate, citrate, and itaconate, which shape HIF-1alpha signaling, inflammatory cytokine production, antimicrobial tolerance, and macrophage polarization. Conversely, PDC activation through thiamine-dependent enzymatic support or pyruvate dehydrogenase kinase inhibition, such as dichloroacetate in experimental models, can improve mitochondrial respiration, ATP synthesis, redox balance, and immune-cell function [48,49]. These findings suggest that future mitochondrial therapy should not focus only on antioxidant delivery but should also consider restoration of pyruvate entry into mitochondria and correction of the lactate-PDC axis in selected patients.

### 6.2. Mitochondria as Immune-System Watchtowers: Bacterial Metabolites, mtROS, and NETs

Recent work extends the concept of mitochondrial immunometabolism by showing that mitochondria can operate as sensory organelles or “watchtowers” of the immune system. In neutrophils infected with Staphylococcus aureus, bacterial lactate can accumulate in phagosomes and be transferred to nearby mitochondria, where it is converted into pyruvate and promotes mitochondrial superoxide generation. This mitochondrial ROS signal acts as a trigger for NETosis, linking detection of bacterial metabolic activity to release of neutrophil extracellular traps [50]. This mechanism is relevant to sepsis because it provides a direct route by which pathogen-derived metabolites can be sensed by host mitochondria and translated into antimicrobial but potentially tissue-damaging responses.

This watchtower function refines the interpretation of NET formation in sepsis. NETs may contribute to bacterial containment, but excessive or persistent NETosis promotes endothelial injury, platelet activation, microvascular thrombosis, histone-mediated cytotoxicity, and organ failure [38,39,50]. Therefore, mitochondrial metabolism in neutrophils should be viewed as a decision node that integrates bacterial metabolites, host lactate-pyruvate handling, mitochondrial ROS, calcium flux, and mtDAMP signaling. Therapeutically, indiscriminate suppression of mitochondrial ROS could weaken pathogen control, whereas timed modulation of excessive mtROS, NETosis, or downstream immunothrombosis may reduce organ injury after infection control has been achieved.

## 7. Organ-Specific Consequences of Mitochondrial Dysfunction in Sepsis

Mitochondrial dysfunction is a systemic process in sepsis, but organ injury is expressed through tissue-specific vulnerabilities. Differences in baseline energy demand, mitochondrial density, substrate preference, antioxidant capacity, immune-cell composition, microvascular architecture, and regenerative potential shape how mitochondrial injury appears in each organ.

The link between immune metabolism and organ dysfunction is mediated by both circulating immune cells and tissue-resident cells. Monocytes and macrophages with a glycolytic, succinate-rich, or PDC-inhibited phenotype can amplify cytokine release, nitric oxide production, and endothelial activation, thereby disturbing microvascular perfusion and mitochondrial substrate delivery in organs. Neutrophil mitochondrial ROS and NETosis connect bacterial sensing to immunothrombosis, capillary plugging, and barrier injury. T-cell metabolic exhaustion contributes to impaired pathogen clearance and secondary infections, which prolong inflammatory and mitochondrial stress. Thus, organ-specific mitochondrial dysfunction emerges from a bidirectional loop: immune-cell metabolic reprogramming injures tissues, and injured tissues release mtDAMPs and metabolites that further reprogram immune cells.

In the heart, sepsis-induced cardiomyopathy is characterized by reversible myocardial depression, altered contractility, impaired beta-adrenergic responsiveness, calcium handling abnormalities, nitric oxide signaling, mitochondrial ROS, and reduced oxidative phosphorylation. Cardiac mitochondria are essential for continuous ATP generation, and even modest bioenergetic impairment can reduce contractile reserve. Mitochondrial permeability transition, mtROS, and impaired mitophagy may contribute to myocardial stunning without extensive necrosis [51]. In the heart, immunometabolic stress interacts with the high ATP demand of cardiomyocytes. Cytokine- and nitric oxide-mediated respiratory inhibition, impaired PDC-dependent pyruvate oxidation, mtROS, and calcium mishandling reduce contractile reserve. At the same time, neutrophil-derived NETs and endothelial activation can impair the coronary microcirculation, creating a mismatch between substrate delivery and mitochondrial utilization.

In the lung, sepsis can cause acute lung injury and acute respiratory distress syndrome through endothelial and epithelial barrier disruption, inflammatory cell infiltration, oxidative stress, and microvascular dysfunction. Mitochondrial injury in alveolar epithelial cells, endothelial cells, and alveolar macrophages may impair barrier repair, increase apoptosis or pyroptosis, and amplify cytokine production. Because the lung is often both a source and target of infection, mitochondrial dysfunction in pulmonary immune cells may shape both pathogen clearance and inflammatory injury [15]. In the lung, immune metabolism influences both barrier failure and host defense. Alveolar macrophage glycolytic activation supports cytokine production, whereas mitochondrial dysfunction in epithelial and endothelial cells impairs barrier repair. Excessive NETosis and mtDAMP-driven inflammasome activation can worsen alveolar-capillary leakage and immunothrombosis, especially when bacterial clearance and inflammatory resolution are uncoupled.

In the kidney, septic acute kidney injury frequently occurs despite variable or only modest reductions in renal blood flow, suggesting that inflammation, microcirculatory dysfunction, tubular stress, and mitochondrial impairment are central. Proximal tubular cells have high energy requirements and depend heavily on mitochondrial oxidative metabolism. Sepsis-associated mitochondrial fragmentation, oxidative injury, altered fatty acid oxidation, mtDNA release, and impaired biogenesis may contribute to tubular dysfunction, inflammatory signaling, and delayed renal recovery [52]. In the kidney, proximal tubular cells are highly dependent on oxidative metabolism and fatty-acid oxidation. A shift toward glycolysis, impaired PDC activity, mitochondrial fragmentation, and insufficient biogenesis may initially conserve energy but can later impair tubular transport and repair. Circulating mtDNA, cytokines, and NET-associated microvascular injury further connect immune-cell metabolism to septic acute kidney injury.

In the brain, sepsis-associated encephalopathy involves neuroinflammation, blood–brain barrier dysfunction, microglial activation, neurotransmitter imbalance, and mitochondrial stress. Neurons are highly dependent on mitochondrial ATP and calcium buffering. Mitochondrial DAMPs and systemic cytokines may activate neuroimmune pathways, while impaired mitophagy and oxidative stress may contribute to cognitive dysfunction during and after critical illness [53]. In the brain, systemic immunometabolism is translated through endothelial activation, blood–brain barrier dysfunction, microglial metabolic reprogramming, and mitochondrial injury in neurons and glia. Multi-omics analyses of sepsis-associated encephalopathy have begun to identify mitochondrial immunometabolic subtypes associated with inflammatory pathways, DAMP signaling, and prognosis, illustrating how organ-specific mitochondrial phenotyping may complement peripheral biomarkers [54].

Skeletal muscle and diaphragm dysfunction are clinically important but often underemphasized consequences of sepsis. Mitochondrial dysfunction, proteolysis, impaired biogenesis, oxidative stress, and reduced contractile protein integrity contribute to ICU-acquired weakness, ventilator weaning difficulty, and long-term functional impairment [30,55]. This is particularly relevant for survivorship, because mitochondrial recovery may influence fatigue, rehabilitation potential, and quality of life after discharge.

Because organ-specific mitochondrial assessment is difficult in critically ill patients, blood-derived biomarkers and circulating immune-cell assays are attractive. However, mitochondrial behavior in peripheral blood mononuclear cells (PBMCs) or platelets may not fully represent mitochondrial function in the heart, kidney, lung, brain, or skeletal muscle. This mismatch should be explicitly considered when designing translational studies and interpreting biomarker data.

## 8. Mitochondrial Biomarkers and Translational Assessment

The clinical translation of mitochondrial biology requires feasible biomarkers. Candidate biomarkers should help identify patients with mitochondrial dysfunction, stratify biological phenotypes, monitor response to therapy, and predict organ recovery or deterioration. Current candidates include circulating mtDNA, oxidized mtDNA, lactate kinetics, oxidative stress markers, peripheral blood mononuclear cell (PBMC) or monocyte respiration, platelet mitochondrial oxygen consumption, mitochondrial membrane potential assays, OXPHOS protein expression, mtDNA copy number, and gene-expression signatures related to biogenesis or mitophagy [16,17]. Candidate mitochondrial biomarkers and assays for translational sepsis studies are summarized in Table 2.

Circulating mtDNA is attractive because it can act as both biomarker and mediator. Elevated mtDNA may reflect tissue injury, impaired mitochondrial clearance, cell death, or immune-cell activation, and can activate TLR9, NLRP3, and cGAS-STING pathways. However, circulating mtDNA assays vary in sample type, processing, target genes, normalization methods, and whether cell-free, vesicle-associated, or cellular mtDNA is measured. Without standardization, it is difficult to compare studies or define clinically actionable thresholds.

Cellular respirometry provides more direct functional information. peripheral blood mononuclear cells (PBMCs), monocytes, lymphocytes, and platelets are accessible from peripheral blood and can be studied using high-resolution respirometry or extracellular flux analysis. These assays may quantify basal respiration, ATP-linked respiration, maximal respiration, spare respiratory capacity, proton leak, and non-mitochondrial oxygen consumption. Recent literature emphasizes monocytes and lymphocytes as practical matrices because they are routinely obtained, immune-relevant, and mitochondria-rich [16]. A 2026 prospective observational study reported increased PBMC respiratory parameters during the first ICU week in sepsis patients compared with matched controls, and found that a progressive increase in basal respiration was associated with 3-month mortality, although the authors interpreted this as exploratory [17]. This illustrates that blood-cell mitochondrial respiration may capture immune-cell activation or stress rather than simple failure.

Platelets are another promising matrix because they contain functional mitochondria, participate in immune-thrombotic responses, and are readily available. Platelet mitochondrial oxygen consumption has been associated in some studies with severity of illness, organ failure, and mortality. However, platelet activation, storage time, medications, transfusion, thrombocytopenia, and analytic conditions can influence results. As with PBMCs, standardization is essential before platelet respiration can become a routine clinical biomarker.

Traditional markers such as lactate remain important but nonspecific. Hyperlactatemia may reflect tissue hypoperfusion, adrenergic stimulation, impaired pyruvate oxidation, mitochondrial dysfunction, altered hepatic clearance, or accelerated glycolysis. Serial lactate measurement is recommended in sepsis resuscitation, but lactate should not be interpreted as a pure mitochondrial biomarker [5]. Combining lactate kinetics with mitochondrial readouts may provide a more nuanced view of bioenergetic stress.

**Figure 4:**
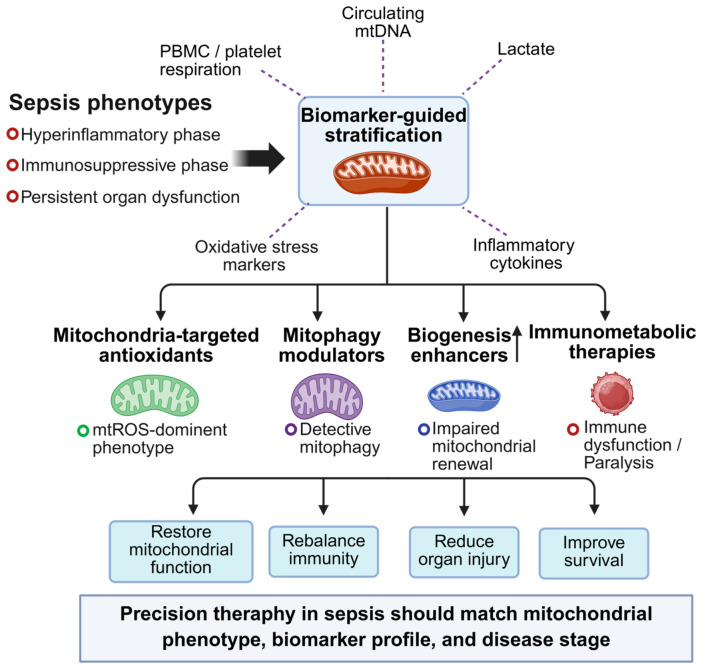
Translational roadmap for biomarker-guided mitochondrial therapy in sepsis. Because sepsis is biologically heterogeneous, mitochondrial interventions should not be applied uniformly. Clinical phenotype, disease stage, organ dysfunction pattern, and mitochondrial biomarkers may help identify patients with predominant oxidative stress, defective mitophagy, impaired mitochondrial biogenesis, or immunometabolic dysfunction. Such stratification may enable more rational selection of mitochondria-targeted therapies. The figure was created with BioRender.com (accessed on 25 May 2026).

The next step is not simply to discover more biomarkers, but to integrate them into therapeutic decision-making. A useful mitochondrial biomarker panel might distinguish patients with mtROS-dominant injury, impaired mitophagy, inadequate biogenesis, immune-cell metabolic paralysis, or persistent DAMP signaling. Figure 4 presents a translational roadmap in which sepsis phenotype, disease stage, and mitochondrial biomarkers are integrated to guide selection of mitochondria-targeted antioxidants, mitophagy modulators, biogenesis enhancers, or immunometabolic therapies.

## 9. Therapeutic Strategies: From Nonspecific Antioxidants to Mitochondria-Targeted Interventions

Mitochondrial dysfunction is an appealing therapeutic target because it lies downstream of diverse septic insults and upstream of organ failure, immune dysfunction, and inflammatory amplification. However, therapeutic translation has been difficult. Sepsis is heterogeneous, mitochondrial dysfunction is dynamic, and the same pathway may be protective at one time point and harmful at another. Therefore, mitochondrial therapy should be considered as an adjunct to, not a replacement for, standard sepsis care. Major therapeutic strategies targeting mitochondrial dysfunction in sepsis are summarized in Table 3.

A more clinically useful way to discuss mitochondria-targeted therapy is to separate agents with direct human sepsis data from those that remain extrapolated from preclinical sepsis or non-sepsis mitochondrial disease trials. MitoQ currently has the most direct human sepsis evidence: a pilot double-blind randomized study in septic shock tested MitoQ 20 mg twice daily for 5 days and showed improvement in oxidative stress biomarkers, but it was not powered to demonstrate significant improvement in mortality, organ recovery, or organ-support outcomes [58]. In contrast, MitoTEMPO, SkQ1, mitoVitE, urolithin A, spermidine, dichloroacetate, and AMPK/SIRT/PGC-1alpha modulators have mechanistic or preclinical support in sepsis-related pathways but require careful clinical translation. Elamipretide is clinically advanced in mitochondrial and cardiolipin-related diseases and is mechanistically relevant to cardiolipin stabilization and cristae integrity, but direct sepsis trials remain lacking [63,64].

Based on current evidence, near-term translational strategies should include: (i) enrichment of patients with mtROS-dominant phenotypes for mitochondria-targeted antioxidants; (ii) testing MitoQ or related compounds with pharmacokinetic and target-engagement endpoints; (iii) combining antioxidants with mitophagy or biogenesis support when biomarkers show both oxidative injury and defective mitochondrial renewal; (iv) evaluating PDC-directed metabolic support, such as thiamine sufficiency or PDK inhibition, in patients with persistent lactate elevation plus evidence of mitochondrial pyruvate oxidation failure; and (v) pairing mtDAMP-pathway inhibitors with biomarkers of persistent mtDNA release, inflammasome activation, or cGAS-STING signaling. These approaches should be adjunctive to source control, antimicrobials, hemodynamic resuscitation, and organ support rather than replacements for standard care.

Conventional antioxidant strategies include vitamin C, N-acetylcysteine, selenium, vitamin E, and melatonin. These agents are biologically plausible because sepsis is associated with oxidative stress and depletion of antioxidant defenses. However, clinical results have been inconsistent. The LOVIT randomized trial found that high-dose intravenous vitamin C in adults with sepsis receiving vasopressors was associated with a higher risk of death or persistent organ dysfunction at 28 days compared with placebo [56]. The 2026 Surviving Sepsis Campaign guidelines suggest against using intravenous vitamin C in adults with sepsis or septic shock, with low certainty of evidence [5]. This does not prove that redox biology is irrelevant; rather, it indicates that nonspecific antioxidant supplementation is unlikely to be sufficient when applied uniformly to unselected patients.

Several factors may explain these failures. First, antioxidant effects are compartmentalized, and systemic antioxidants may not adequately reach mitochondria. Second, timing matters: ROS participate in antimicrobial defense and adaptive signaling early in infection, whereas persistent mtROS may become pathological later. Third, dosing may be either insufficient to affect mitochondrial redox stress or excessive in ways that disrupt beneficial signaling. Fourth, clinical sepsis phenotypes differ: some patients may have dominant oxidative injury, while others have immunosuppression, endothelial dysfunction, coagulopathy, or impaired biogenesis. Fifth, redox biomarkers are rarely used to select patients or confirm target engagement.

Mitochondria-targeted antioxidants were designed to overcome some of these limitations. MitoQ couples a ubiquinone antioxidant moiety to a lipophilic triphenylphosphonium cation, promoting accumulation within the mitochondrial matrix driven by membrane potential. MitoTEMPO targets a nitroxide antioxidant to mitochondria. SkQ1, mitoVitE, and related compounds also aim to concentrate antioxidant activity within mitochondria [45,46,57,60,61,62,63,65]. In preclinical sepsis models, mitochondria-targeted antioxidants have reduced mitochondrial oxidative damage, inflammatory cytokines, biochemical markers of organ injury, cardiac dysfunction, renal dysfunction, and diaphragm weakness [55,59,60,61,62].

Clinical evidence remains limited but is emerging. A pilot double-blind randomized trial of MitoQ in 42 patients with septic shock evaluated SOFA trajectory and oxidative stress biomarkers. MitoQ improved several oxidative biomarkers at day 5 compared with placebo, but significant differences were not observed in 28-day mortality, organ recovery, or organ support, and larger adequately powered trials are needed [58]. This result is scientifically informative: it suggests that mitochondrial target engagement may be measurable, but biomarker improvement alone does not guarantee clinical benefit. Future trials should incorporate patient selection, timing, pharmacokinetics, mitochondrial biomarkers, and organ-specific outcomes.

Another therapeutic strategy is modulation of mitochondrial quality control. Enhancing mitophagy may reduce accumulation of damaged mitochondria, decrease mtDNA release, and limit inflammasome activation. AMPK activators, SIRT modulators, urolithin A, spermidine, resveratrol, metformin, and other agents have been discussed in relation to mitochondrial homeostasis, although most evidence in sepsis remains preclinical or indirect. These approaches must be evaluated carefully because excessive mitophagy without adequate biogenesis may reduce mitochondrial mass and worsen bioenergetic capacity.

Stimulating mitochondrial biogenesis is also attractive. PGC-1alpha-related programs, NRF1/NRF2, TFAM, SIRT1/SIRT3, and AMPK pathways coordinate mitochondrial recovery. Enhancing these pathways could support tissue repair, immune recovery, and post-sepsis rehabilitation. However, biogenesis is energy-demanding and may not be beneficial during uncontrolled infection or unresolved shock. Again, timing and phenotype are crucial.

Mitochondrial membrane stabilization and cardiolipin-targeted therapies represent another avenue. Peptides such as elamipretide have been investigated in mitochondrial diseases and ischemia–reperfusion contexts because of effects on cardiolipin interactions, cristae structure, and respiratory-chain efficiency. Although direct clinical evidence in sepsis remains insufficient, membrane-stabilizing approaches are conceptually relevant because mitochondrial permeability transition, cristae remodeling, and cardiolipin externalization contribute to sepsis-related mitochondrial injury and DAMP signaling.

Finally, immunometabolic therapies may aim to restore immune-cell mitochondrial competence rather than only reduce oxidative stress. In patients with immune paralysis, interventions that improve mitochondrial fitness in monocytes, dendritic cells, or T cells might enhance pathogen clearance and reduce secondary infection risk. In patients with hyperinflammatory mtDAMP signaling, interventions that reduce mtDNA release, enhance mitophagy, or inhibit downstream pathways such as NLRP3 or cGAS-STING may be more rational. These examples show why mitochondrial therapy should be developed as precision adjunctive therapy.

## 10. Future Perspectives and Conclusions

Future research should move from general mitochondrial concepts to testable clinical programs. First, specific combination strategies should be developed. Examples include pairing mitochondria-targeted antioxidants with mitophagy enhancers in mtROS-high/mitophagy-low phenotypes, combining PDC-directed metabolic support with mitochondrial biogenesis stimulation during recovery, and integrating mtDAMP-pathway modulation with immunothrombosis control in patients with persistent mtDNA release and NET-associated organ injury. Combination therapy should be stage-specific: early treatment should preserve antimicrobial mitochondrial signaling, whereas later treatment may prioritize repair, immune recovery, and organ rehabilitation.

Second, multi-omics approaches should be used to define mitochondrial phenotypes in sepsis patients. Integrating transcriptomics, proteomics, metabolomics, lipidomics, mtDNA measurements, single-cell data, and immune-cell respirometry may identify subgroups such as mtROS-dominant injury, PDC-lactate axis failure, defective mitophagy, impaired biogenesis, persistent mtDAMP signaling, or immune-cell metabolic paralysis. These phenotypes should be linked to organ-specific outcomes rather than only mortality.

Third, standardized mitochondrial function assessment is needed for clinical research and eventual practice. Minimum reporting standards should include sample type, timing from sepsis onset, anticoagulant, processing interval, cell isolation method, respirometry platform, normalization strategy, respiratory states measured, mtDNA target genes, pre-analytical hemolysis control, and calibration procedures. Without standardization, mitochondrial biomarkers will remain difficult to compare across studies and impossible to convert into actionable thresholds.

Fourth, artificial intelligence and machine learning should be applied to mitochondrial biomarker data, but with clinically transparent validation. Algorithms could integrate lactate kinetics, circulating mtDNA, PBMC or platelet respiration, redox markers, inflammatory cytokines, routine laboratory data, organ-support variables, and omics features to predict mitochondrial phenotypes and treatment response. Such models should be externally validated, interpretable, and embedded in prospective trials rather than used only for retrospective clustering [54].

This review also has several limitations. First, many mechanistic insights are derived from experimental endotoxemia or animal sepsis models and may not fully reproduce the heterogeneity, comorbidities, timing, and treatment effects observed in human sepsis. Second, blood-based mitochondrial biomarkers, including circulating mtDNA and PBMC or platelet respiration, are clinically feasible but may not directly reflect mitochondrial dysfunction in inaccessible organs such as the heart, kidney, lung, brain, or skeletal muscle. Third, differences in sampling time, pre-analytical handling, assay platforms, and normalization methods still limit comparison across studies. Finally, most mitochondria-targeted therapies remain supported mainly by preclinical or early-phase clinical evidence; therefore, future trials should incorporate standardized mitochondrial endpoints and biomarker-guided patient selection.

In conclusion, mitochondria occupy a central position at the intersection of oxidative stress, immune dysregulation, inflammatory signaling, and organ failure in sepsis. Septic mitochondrial dysfunction is not merely a passive consequence of inflammation; it is an active process that can amplify host injury through bioenergetic failure, mitochondrial DAMP release, inflammasome activation, and immunometabolic disruption. The next generation of sepsis therapeutics should move beyond nonspecific antioxidant supplementation toward mitochondria-targeted, time-sensitive, and biomarker-guided strategies. Such an approach will require integration of mechanistic biology with clinically feasible assays and patient-centered outcomes. Addressing the reviewer-identified gaps requires mechanistic integration of immunometabolism, mtDAMP clinical relevance, organ-specific mitochondrial phenotypes, and trial-ready therapeutic strategies. A precision mitochondrial medicine framework for sepsis should therefore combine serial biomarkers, organ-specific outcomes, and staged interventions that restore mitochondrial function without compromising host defense.

## Figures and Tables

**Table 1:** Major mitochondrial mechanisms in sepsis.

Mechanism	Key Molecular Features	Pathophysiological Relevance in Sepsis	References
Impaired OXPHOS	ETC complex dysfunction; reduced coupling; ATP synthase impairment	Bioenergetic failure, impaired oxygen utilization, cytopathic hypoxia, and organ dysfunction despite resuscitation	[10,11,12,13,14,15]
mtROS/RNS excess	Superoxide, H_2_O_2_, nitric oxide, peroxynitrite	Oxidative damage to proteins, lipids, mtDNA, and ETC complexes; redox phenotype	[13,14,18,19,20,21]
Calcium dysregulation	Mitochondrial Ca^2+^ overload; mPTP opening	Membrane potential loss, impaired ATP production, and apoptosis	[11,13,15,27]
Mitochondrial dynamics imbalance	DRP1-mediated fission; altered MFN1/2 and OPA1 fusion	Fragmentation or maladaptive remodeling; altered recovery kinetics	[22,23,24,25]
Defective mitophagy	PINK1/Parkin, BNIP3/NIX, impaired autophagosome-lysosome flux	Accumulation of damaged mitochondria and increased mtDAMP release	[15,22,23,24,25,26,28,29]
Insufficient biogenesis	AMPK-SIRT-PGC-1alpha-NRF-TFAM axis	Failure to restore mitochondrial mass and function; prolonged organ dysfunction	[25,26,30]
mtDAMP release	mtDNA, oxidized mtDNA, cardiolipin, ATP, N-formyl peptides	Activation of TLR9, NLRP3, cGAS-STING, neutrophils, endothelium, and immunothrombosis	[28,29,31,32,33,34,35,36,37,38,39]
Immunometabolic failure	Altered macrophage, neutrophil, and T-cell mitochondrial metabolism	Hyperinflammation, immune paralysis, and impaired pathogen clearance	[40,41,42,43,44]

Abbreviations: ETC, electron transport chain; mPTP, mitochondrial permeability transition pore; mtDAMP, mitochondrial damage-associated molecular pattern; mtDNA, mitochondrial DNA; mtROS, mitochondrial reactive oxygen species; OXPHOS, oxidative phosphorylation; RNS, reactive nitrogen species.

**Table 2:** Candidate mitochondrial biomarkers for translational sepsis studies.

Biomarker or Assay	Sample or Method	Clinical Utility and Key Limitations	References
Circulating mtDNA	Plasma or serum qPCR/ddPCR	Feasible marker of mitochondrial release and DAMP burden; associated with severity/prognosis in some studies, but affected by pre-analytics and cut-offs	[16,32,33]
Oxidized mtDNA	DNA damage or 8-oxo-dG assays	Closer link to immunostimulatory mtDNA; technically demanding and less validated	[28,29]
PBMC respiration	High-resolution respirometry or extracellular flux analysis	Serial immune-cell bioenergetics; influenced by cell mixture and disease phase	[16,17]
Monocyte/lymphocyte respiration	Cell sorting plus respirometry	Cell-specific immunometabolic phenotype; requires rapid processing and expertise	[16,17,40]
Platelet oxygen consumption	Platelet respirometry	Accessible mitochondrial/immunothrombotic readout; affected by activation, drugs, and storage	[16]
Lactate kinetics	Routine clinical blood test	Clinically established marker of stress and perfusion; useful serially but not specific to mitochondria	[5,6]
Redox markers	MDA, protein carbonyls, thiols, SOD, CAT, GPx	Target-engagement markers for oxidative stress; compartment- and timing-dependent	[13,14,18,19,20,27]
Biogenesis/quality-control markers	PGC-1alpha, NRF1/2, TFAM, OXPHOS proteins	May identify mitochondrial repair phenotype; not standardized for ICU use	[15,25,26]

Abbreviations: CAT, catalase; ddPCR, droplet digital PCR; GPx, glutathione peroxidase; ICU, intensive care unit; MDA, malondialdehyde; mtDNA, mitochondrial DNA; PBMC, peripheral blood mononuclear cell; qPCR, quantitative polymerase chain reaction; SOD, superoxide dismutase.

**Table 3:** Therapeutic strategies targeting mitochondrial dysfunction in sepsis.

Strategy	Representative Examples	Rationale and Translational Status	References
Conventional antioxidants	Vitamin C, NAC, selenium, vitamin E, melatonin	Reduce systemic oxidative stress; mixed clinical evidence; routine IV vitamin C is not recommended	[5,56,57]
Mitochondria-targeted antioxidants	MitoQ, MitoTEMPO, SkQ1, mitoVitE	Concentrate antioxidant activity in mitochondria; preclinical benefit; human MitoQ pilot showed biomarker improvement but no definitive clinical outcome benefit	[55,58,59,60,61,62,63,64,65]
Membrane/cardiolipin stabilization	SS-31/elamipretide-like approaches	Improve cristae integrity, membrane stability, and respiratory efficiency; direct sepsis evidence limited	[63,64]
Mitophagy modulation	AMPK activation, PINK1/Parkin strategies, urolithin A, spermidine	Remove damaged mitochondria and reduce DAMP release; excessive mitophagy may reduce mitochondrial mass	[22,23,24,25,26]
Biogenesis enhancement	PGC-1alpha, SIRT1/3, NRF/TFAM pathways	Restore mitochondrial mass and oxidative capacity; timing and energy demand are key limitations	[15,25,26]
Immunometabolic therapy	Stage-specific macrophage, monocyte, or T-cell metabolic modulation	Restore immune competence or limit hyperinflammation; requires immune and metabolic phenotyping	[40,41,42,43,44]
DAMP pathway targeting	TLR9, NLRP3, cGAS-STING modulation	Block inflammatory consequences of mitochondrial damage; investigational and may impair host defense if mistimed	[28,29,31,32,33,34,35,36,37]

Abbreviations: AMPK, AMP-activated protein kinase; DAMP, damage-associated molecular pattern; NAC, N-acetylcysteine.

## Data Availability

No new data were created or analyzed in this study. Data sharing is not applicable to this article.
